# Cross-Reactivity of Anthrax and C2 Toxin: Protective Antigen Promotes the Uptake of Botulinum C2I Toxin into Human Endothelial Cells

**DOI:** 10.1371/journal.pone.0023133

**Published:** 2011-08-05

**Authors:** Angelika Kronhardt, Monica Rolando, Christoph Beitzinger, Caroline Stefani, Michael Leuber, Gilles Flatau, Michel R. Popoff, Roland Benz, Emmanuel Lemichez

**Affiliations:** 1 Rudolf-Virchow-Center, University of Würzburg, Würzburg, Germany; 2 Inserm, U895, Toxines Microbiennes dans la Relation Hôte-Pathogènes, Batiment Archimed, Nice, France; 3 Faculté de Médecine, Institut Fédératif de Recherche 50, Université de Nice-Sophia Antipolis, Nice, France; 4 Unité des Bactéries Anaerobies et Toxines, Institut Pasteur, Paris, France; 5 School of Engineering and Science, Jacobs University Bremen, Bremen, Germany; 6 Laboratoire central de bactériologie, Centre Hospitalier Universitaire de Nice, Nice, France; University of Edinburgh, United Kingdom

## Abstract

Binary toxins are among the most potent bacterial protein toxins performing a cooperative mode of translocation and exhibit fatal enzymatic activities in eukaryotic cells. Anthrax and C2 toxin are the most prominent examples for the AB_7/8_ type of toxins. The B subunits bind both host cell receptors and the enzymatic A polypeptides to trigger their internalization and translocation into the host cell cytosol. C2 toxin is composed of an actin ADP-ribosyltransferase (C2I) and C2II binding subunits. Anthrax toxin is composed of adenylate cyclase (EF) and MAPKK protease (LF) enzymatic components associated to protective antigen (PA) binding subunit. The binding and translocation components anthrax protective antigen (PA_63_) and C2II of C2 toxin share a sequence homology of about 35%, suggesting that they might substitute for each other. Here we show by conducting *in vitro* measurements that PA_63_ binds C2I and that C2II can bind both EF and LF. Anthrax edema factor (EF) and lethal factor (LF) have higher affinities to bind to channels formed by C2II than C2 toxin's C2I binds to anthrax protective antigen (PA_63_). Furthermore, we could demonstrate that PA in high concentration has the ability to transport the enzymatic moiety C2I into target cells, causing actin modification and cell rounding. In contrast, C2II does not show significant capacity to promote cell intoxication by EF and LF. Together, our data unveiled the remarkable flexibility of PA in promoting C2I heterologous polypeptide translocation into cells.

## Introduction

Binary toxins of the AB_7/8_ type are highly potent and specialized bacterial protein toxins and are organized in two different polypeptide chains that are separately secreted in the external media of Gram-positive bacteria [1]. Component A is responsible for the intracellular enzymatic activity of the toxin, whereas heptamers or octamers, of the component B are necessary for receptor-binding and translocation of component A into target cells. Given the close homology of structure of the binding components of these two-component toxins it is of importance to decipher whether each component can functionally substitute for each other to intoxicate cells, that we termed cross-reactivity.

One of the most prominent toxins of this type of toxin is anthrax toxin from *Bacillus anthracis* [2]. This toxin possesses a binding and translocation component, protective antigen (PA) and two enzymatic subunits, edema factor (EF) and lethal factor (LF). Edema factor (EF) is an 89 kilo Dalton Ca^2+^- and calmodulin-dependent adenylate cyclase which catalyzes the production of intracellular cAMP and causes severe edema. Lethal factor (LF) is a 90 kilo Dalton Zn^2+^-binding metalloprotease that cleaves mitogen-activated protein kinase kinases (MAPK-kinases) and thereby interferes with the MAPK cascade, a major signaling pathway, triggered by surface receptors, controlling cell proliferation and survival [3], [4], [5]. The binding component PA is essential for delivery of both enzymes into the target cells [3], [6], [7]. It is secreted as an 83 kilo Dalton water-soluble precursor form (PA_83_) and needs to undergo proteolytic activation by cell-bound furin. After the activation of PA_83_, the remaining 63 kilo Dalton PA_63_ forms an oligomeric channel responsible for the binding and translocation of EF and/or LF into the cytosol of target cells [8], [9], [10], [11], [12].

*Clostridium botulinum*, well known for the production of potent neurotoxins, produces various protein toxins, such as the AB type C2 toxin [13], [14], [15]. The binding component of C2 toxin, C2II (60 kilo Dalton after proteolytic cleavage with trypsin), forms heptamers that insert into biological and artificial membranes at an acidic pH and promotes the translocation of the 45 kilo Dalton enzymatic component C2I into the cytosol of the target cells upon receptor-mediated endocytosis of the complex [16], [17]. C2I acts as an ADP-ribosyltransferase on monomeric G-actin, causing disruption of the actin cytoskeleton [18], [19].

The enzymatic components of anthrax and C2 toxin differ significantly in their enzymatic activity and do not show any homology in their primary structures. However, the binding components PA and C2II share a considerable sequence homology of about 35% in two of three domains, indicating that they are closely related in structure and hence also in function [3], [8], [20], [21]. In recent years, many important structural features, particularly concerning PA, have been unveiled, such as the Φ-clamp and the loop network responsible for allocation of the PA monomers [22], [23]. Interesting details concerning the possible mode of translocation are known, all favoring an acid-induced disassembly of the enzymatic components to a molten globular state, followed by threading of the N-terminal part of the polypeptide chain through the pore [22], [23], [24], [25]. However, the exact mode of transporting the enzymatic components into the cytosol of target cell is still not fully solved. The first crucial step of the translocation mechanism is the binding of the enzymatic components to the receptor-bound prepore on the cell surface [1]. Previous results of our and other groups evidenced that truncated forms of the enzymatic components as well as full size EF and LF block the pores formed by PA_63_, and that an N-terminal His_6_-tag strengthens their affinity [20], [26], [27]. Binding of the N-terminal ends of EF and LF to PA_63_ is followed by endocytosis, acidification of the endosomes and finally release of the enzymatic components into the cytosol of target cells, where they exert their fatal enzymatic activities [9], [10], [28]. Interestingly, LF's amino-terminal part, LF_N_ (LF_1-254_), is sufficient to confer the ability to associate with PA_63_ pores. It can even be used to drive the translocation of unrelated polypeptides into target cells via PA_63_ or C2II [29], [30].

To further elucidate the mode of binary toxins' translocation into target cells and the possible cross-reactivity of the different enzymatic components via the homologous binding component of the other toxin; we performed *in vitro* and *in vivo* (i.e. cell-based assay) experiments interchanging the different A-B components of anthrax and C2 toxin. Most importantly our data show the high capacity of PA_63_ to bind C2I *in vitro* in the black lipid bilayer assay. Complementary to these findings we evidence the functionality of PA/C2I chimera toxin combination in cell intoxication. Further, C2II appeared more specifically involved in C2I binding and translocation. Together, our data unveiled the remarkable ability of PA to support cell intoxication by C2I, a distantly related AB_7/8_ toxin component.

## Materials and Methods

### Materials

PA, LF and EF genes were PCR-amplified from genomic DNA of *Bacillus anthracis* strain Sterne (a kind gift of Patrice Boquet, Nice, France) and cloned into the pQE30 (Qiagen), pET28a and pET22b (Novagen) expression plasmids, respectively. The N-terminal His_6_-tag was removed from His_6_-EF by incubation with thrombin and from His_6_-LF with enteropeptidase, respectively. Nicked anthrax PA_63_ from *B. anthracis* was obtained from List Biological Laboratories Inc., Campbell, CA. One mg of lyophilized protein was dissolved in 1 ml 5 mM HEPES, 50 mM NaCl, pH 7.5 complemented with 1.25% trehalose. Aliquots were stored at −20°C. Channel formation by PA_63_ was stable for months under these conditions. C2I and C2II genes were PCR-amplified from genomic DNA of *Clostridium botulinum* D strain 1873 and cloned into pET22 (Novagen) and pQE30 (Qiagen) expression plasmids. All genes were cloned with *Bam*HI-*Sac*I restriction sites. Recombinant toxins containing His_6_-tags were expressed in *Escherichia. coli* BL21 (DE3) and purified on a Chelating Sepharose Fast Flow column previously chelated with nickel (Amersham Biosciences) as recommended by the manufacturer and described previously [5]. Fractions containing toxin were pooled and dialyzed over night against 250 mM NaCl and 25 mM Tris-HCl, pH 8. Recombinant C2II and C2I proteins used for bilayer measurements were cloned in pGEX-2T vector in *E. coli* BL21 cells and expressed as glutathione *S*-transferase (GST) fusion proteins with the glutathione *S*-transferase-fusion Gene Fusion System from Amersham Pharmacia Biotech [17], [18]. The proteins were purified as described previously [18] and incubated with thrombin (3.25 NIH units/ml bead suspension) for cleavage of the GST-tag [31]. C2II was activated with 0.2 µg of trypsin per microgram of protein for 30 min at 37°C [31].

### Western Blots

The polyclonal antibodies against the N-terminal part of MEK2 (N20) were purchased from Santa Cruz Biotechnology; monoclonal antibodies against ß-actin were obtained from Sigma-Aldrich (clone AC-74). Primary antibodies were visualized using goat anti-mouse or anti-rabbit horseradish peroxidase-conjugated secondary antibodies (DakoCytomation), followed by chemiluminiscence detection ECL (GE Healthcare).

### Cell culture

Human umbilical vein endothelial cells (HUVECs, a human primary cell line obtained from PromoCell) were grown in serum-free medium (SFM) supplemented with 20% FBS (Invitrogen), 20 ng/ml basic ßFGF (Invitrogen), 10 ng/ml EGF (Invitrogen) and 1 µg/ml heparin (Sigma-Aldrich) as described previously [32].

### Adenylate cyclase activity

Intracellular concentration of cyclic AMP (cAMP) was determined using the Cyclic AMP Assay (R&D Systems).

### ADP-ribosylation

Control cells or intoxicated HUVECs (10^5^ cells/conditions) were homogenized in 0.25 ml cold BSI buffer (3 mM imidazole pH 7.4, 250 mM sucrose) supplemented additionally with 1 mM phenylmethylsulfonyl fluoride. Cells were lysed by passing through a 1 ml syringe equipped with a 25G ×5/8”-needle (U-100 Insulin, Terumo) 40 times. Nuclei were removed by centrifugation for 10 minutes at 4°C. Protein concentrations of the post-nuclear supernatants were determined using Dc protein assay (Bio-Rad). ADP-ribosylation was performed for 90 minutes at 37°C on 5 µg of intoxicated cell lysates, supplemented with 0.5 µCi [^32^P]-NAD (800 Ci/mmol) and 1 µg of C2I. Proteins were resolved on 12% SDS-PAGE and *in vitro* ADP-ribosylated actin was revealed using a phosphorimaging system.

### Lipid bilayer experiments

Black lipid bilayer measurements were performed as described previously [33]. The instrumentation consisted of a Teflon chamber with two aqueous compartments connected by a small circular hole. The hole had a surface area of about 0.4 mm^2^. Membranes were formed by painting a 1% solution of diphytanoyl phosphatidylcholine (Avanti Polar Lipids, Alabaster, AL) in n-decane onto the hole. The aqueous salt solutions (Merck, Darmstadt, Germany) were buffered with 10 mM MES to pH 5.5 to pH 6. Control experiments revealed that the pH was stable during the time course of the experiments. The binding components of the binary toxins were reconstituted into the lipid bilayer membranes by adding concentrated solutions to the aqueous phase to one side (the *cis*-side) of a black membrane. The temperature was kept at 20°C throughout. Membrane conductance was measured after application of a fixed membrane potential with a pair of silver/silver chloride electrodes inserted into the aqueous solutions on both sides of the membrane. Membrane current was measured using a homemade current-to-voltage converter made with a Burr Brown operational amplifier. The amplified signal was monitored on a storage oscilloscope and recorded on a strip chart recorder.

### Binding experiments

The binding of EF and LF to the C2II channel and the binding of C2I to the PA_63_ and to the C2II channel was investigated performing titration experiments similar to those used previously to study the binding of 4-aminoquinolones to the PA_63_ and C2II channels and LF to the PA_63_ channel in single- or multi-channel experiments [34], [35], [36]. The PA_63_ and C2II channels were reconstituted into lipid bilayers. About 60 minutes after the addition of either activated PA_63_ or C2II to the *cis*-side of the membrane, the rate of channel insertion in the membranes was very small. Then concentrated solutions of EF, LF or C2I were added to the *cis*-side of the membranes while stirring to allow equilibration. The results of the titration experiments, i.e. the blockage of the channels, were analyzed using Langmuir adsorption isotherms (see eqn. [1]) [20], [37].
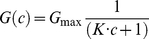
(1)


*G(c)* is the conductance of the channels at a given concentration *c* of the enzymatic components and *G_max_* is their conductance before the start of the titration experiment (at *c* = 0). *K* is the stability constant for binding of the enzymatic components of the binary toxins to the PA_63_ or C2II channels. The half saturation constant *K_s_* of binding is given by the inverse stability constant 1/*K*. The percentage of blocked channels is given by:
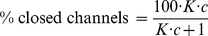
(2)


### Statistics

Unpaired, two-sided Student's t-test was used to analyze biological data with * p<0.05. The statistical software used was Prism 5.0b. The fit of the data from the titration experiments with lipid bilayers was performed using the scientific data graphics application program Fig.P. For most of the fits of the titration data with eqn. [2] we obtained r^2^ >0.99.

## Results

### Interaction of PA_63_ with C2I *in vitro*

**Figure 1:**
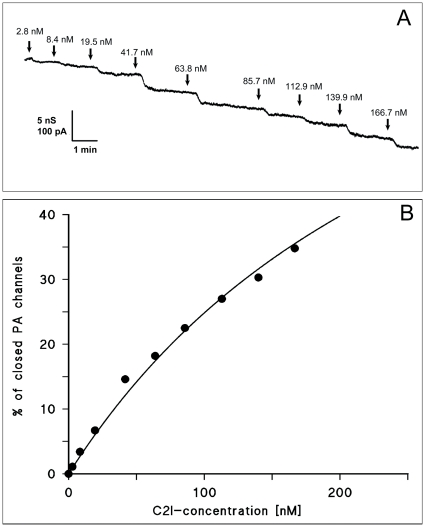
Interaction of C2I with PA_63_ channels. **A**: Titration of PA_63_ induced membrane conductance with C2I. The membrane was formed from diphytanoyl phosphatidylcholine/n-decane, containing about 5,500 channels. C2I was added at the concentrations shown at the top of the panel. Finally, about 40% of the PA_63_ channels were blocked. The aqueous phase contained 1 ng/ml activated PA_63_ protein (added only to the *cis*-side of the membrane), 150 mM KCl, 10 mM MES pH 6. The temperature was 20°C and the applied voltage was 20 mV. Note that C2I only blocks PA_63_ channels when it is added to the *cis*-side of the membrane (data not shown). **B**: Lineweaver-Burke plot of the inhibition of the PA_63_-induced membrane conductance by C2I. The fit was obtained by linear regression of the data points taken from Figure 1A (r^2^ = 0.996654) and corresponds to a stability constant *K* for C2I binding to PA_63_ of (3.98±0.063) ×10^6^ M^−1^ for C2I binding to PA_63_ (half saturation constant K_S_ = 251 nM).

The stability constant *K* for the binding of C2I to the PA_63_ channel was measured in multi-channel experiments, performed as described previously [35]. About 60 minutes after addition of the protein, the rate of conductance increase had slowed down considerably. At that time, small amounts of a concentrated enzyme solution were added to the *cis*-side of the membrane and the PA_63_-induced membrane conductance decreased in a dose-dependent manner. Figure 1A shows an example for a titration experiment with an applied voltage of 20 mV in 150 mM KCl in which increasing concentrations of C2I (arrows) were added to the *cis*-side of a membrane containing about 5,500 PA_63_ channels. The membrane conductance decreased as a function of the C2I concentration within a few minutes after addition of C2I (see figure 1A). The data of Figure 1A and of similar experiments were analyzed using equation [2] as described previously [35], [38]. The plots of the percentage of closed channels as a function of the enzyme concentrations were used to calculate the stability constants *K* for binding as it is shown in Figure 1B for the data of Figure 1A. The fit curve (solid line in Figure 1A) corresponds to a stability constant *K* of (3.98±0.063) ×10^6^ M^−1^ for C2I binding to PA_63_ (half saturation constant K_S_ = 251 nM). The stability constant *K* of the binding of C2I to the PA_63_ channels was averaged out of at least five individual experiments resulting in *K* (5.1±1.5) ×10^6^ M^−1^ (half-saturation constant *K_s_* = 196 nM) (see Table 1). Measurements with artificial bilayer membranes of the wild-type AB components C2II and C2I revealed a stability constant *K* of (3.7±0.4) ×10^7^ M^−1^ with a half saturation constant *K_S_* of 27.2 nM.

**Table 1:** Stability constants *K* and half saturation constants *K_S_* for the cross-reaction of anthrax and C2 toxin.

Toxin combination		*K* [M^−1^]	*K_s_* [nM]
**PA with**			
	C2I	(0.51±0.15) ×10^7^	196
	**EF**	**14.5×10^7^**	**6.9**
	**LF**	**36.2×10^7^**	**2.8**
**C2II with**			
	EF	(7.7±4.8) ×10^7^	13.0
	LF	(2.0±0.3) ×10^7^	49.9
	C2I	(3.7±0.4) ×10^7^	27.2

bility constants *K* for the binding of C2I, EF or LF to PA_63_ or C2II channels reconstituted in lipid bilayer membranes. The membranes were formed from diphytanoyl phosphatidylcholine/n-decane. The aqueous phase contained 150 mM KCl, buffered to pH 5.5 to 6 using 10 mM MES-KOH; T = 20°C. Measurements were performed at a membrane potential of 20 mV. The data represent the means of at least three individual titration experiments. *K_S_* is the half saturation constant, i.e. 1/*K*. Some of the wild-type toxin combinations (given in bold) were taken from reference [20].

### Binding of C2II with EF and LF *in vitro*

As demonstrated in recent studies, EF and LF of anthrax toxin are able to block the PA_63_ pore in artificial bilayer membranes at nanomolar concentrations [20] and C2II channels can be blocked by their enzymatic counterpart C2I [18]. The possible binding of EF and LF to the C2II channels was studied using titration experiments as described above for PA_63_ and C2I shown in Figure 1. These measurements allowed the calculation of the stability constants *K* of EF and LF binding to the C2II channels, resulting in (7.7±4.8) ×10^7^ M^−1^ and (2.0±0.3) ×10^7^ M^−1^, respectively (see Table 1). The data indicated that EF and LF have a high affinity for the C2II channels *in vitro*, as the half saturation constants *K_S_* for EF and LF binding to the C2II channels were 13.0 nM and 49.9 nM, respectively.

### PA_63_ translocates C2I in HUVECs

**Figure 2:**
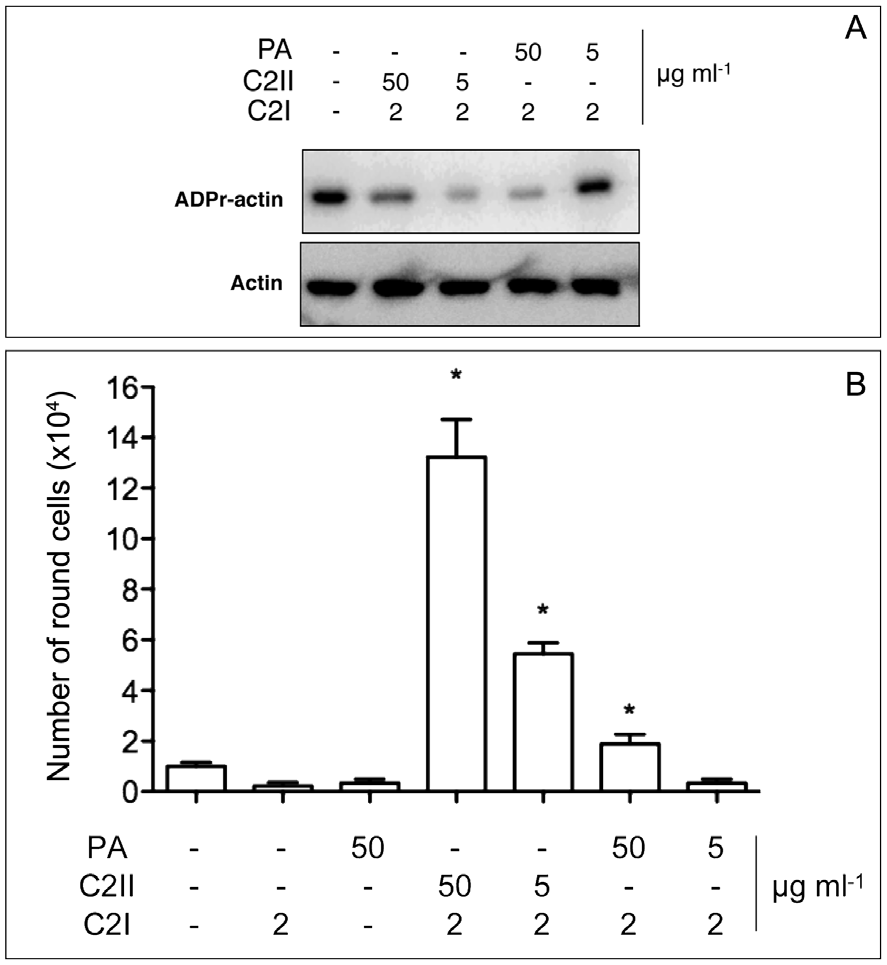
Specificity of HUVECs intoxication by C2I using PA_63_ in comparison to C2II. HUVECs (5×10^5^ cells/100 mm well) were intoxicated with the indicated concentration of polypeptides during 48 (A) or 24 hours (B). **A**: Cells were intoxicated as indicated and levels of cellular ADP-ribosylated actin (ADPr-actin) were determined by *in vitro* ADP-ribosylation of cell lysates with C2I and radiolabeled [^32^P]-NAD. Under these conditions ADP-ribosylated actin formed during the intoxication process is no longer labeled by *in vitro* ADP-ribosylation, which is indicated by decrease of radioactivity. Immunoblotting anti-beta-actin was performed in parallel on cell lysates to show actin protein levels engaged in the ADP-ribosylation experiments. ADP-ribosylation signals were normalized to actin immunoblot signals. **B**: Efficiency of cell intoxication. Cells were intoxicated and the number of round cells was directly assessed by counting floating cells. The columns show mean values of 5 independent counting for the individual conditions ± SEM (ns: non significant; * p<0.05 versus control).

C2I acts as an ADP-ribosyltransferase targeting cellular G-actin. Therefore, successful delivery of this enzymatic component into target cells can be detected by disruption of the cytoskeleton followed by rounding up of target cells and detachment of target cells from the extracellular matrix, defined as intoxicated cells [18] or by direct measurement of the modified G-actin as described in Materials and Methods. C2I, added to different concentrations of its native binding component C2II, led to increasing numbers of round cells after 24 hours of intoxication (data not shown). Figure 2A shows the direct measurement of cellular ADP-ribosylated actin (ADPr-actin) in HUVECs after treatment with different PA-C2I and C2II-C2I combinations. The cells were intoxicated with different concentrations of binding components and effectors as indicated. Levels of ADP-ribosylated actin (ADPr-actin) were determined by *in vitro* ADP-ribosylation of cell lysates with C2I and radiolabeled [^32^P]-NAD. Under these conditions ADP-ribosylated actin produced by the intoxication process is no longer labeled by *in vitro* ADP-ribosylation. The results clearly demonstrated that the radioactivity combined with labeled ADPr-actin decreased for the combinations PA-C2I, when PA was applied in high concentration, and C2II-C2I suggesting that both channels were able to transport C2I into HUVECs. Controls did not reveal any change of the labeling of actin, which means that neither PA nor C2I alone, respectively, modified intracellular actin (data not shown).

Similar results were obtained when the number of intoxicated HUVECs was determined by counting round cells as a result of C2I activity on actin. Figure 2B shows the efficiency of cell intoxication under different experimental conditions. HUVECs were incubated with different combinations of PA-C2I and C2II-C2I as indicated, and the number of intoxicated cells was directly assessed. The results shown in Figure 2B revealed that the number of intoxicated cells was highest for the native combination C2II-C2I. However, when HUVECs were incubated with C2I and different quantities of PA, rounding of cells was detected even at lower probability (Figure 2B). This demonstrated that C2I was transported by PA channels into HUVECs. The results of Figure 2B indicated a dose-dependent process as some combinations failed to induce any significant effect compared to the controls.

### Interaction of C2II with LF and EF *in vivo* (cell-based assay)

**Figure 3:**
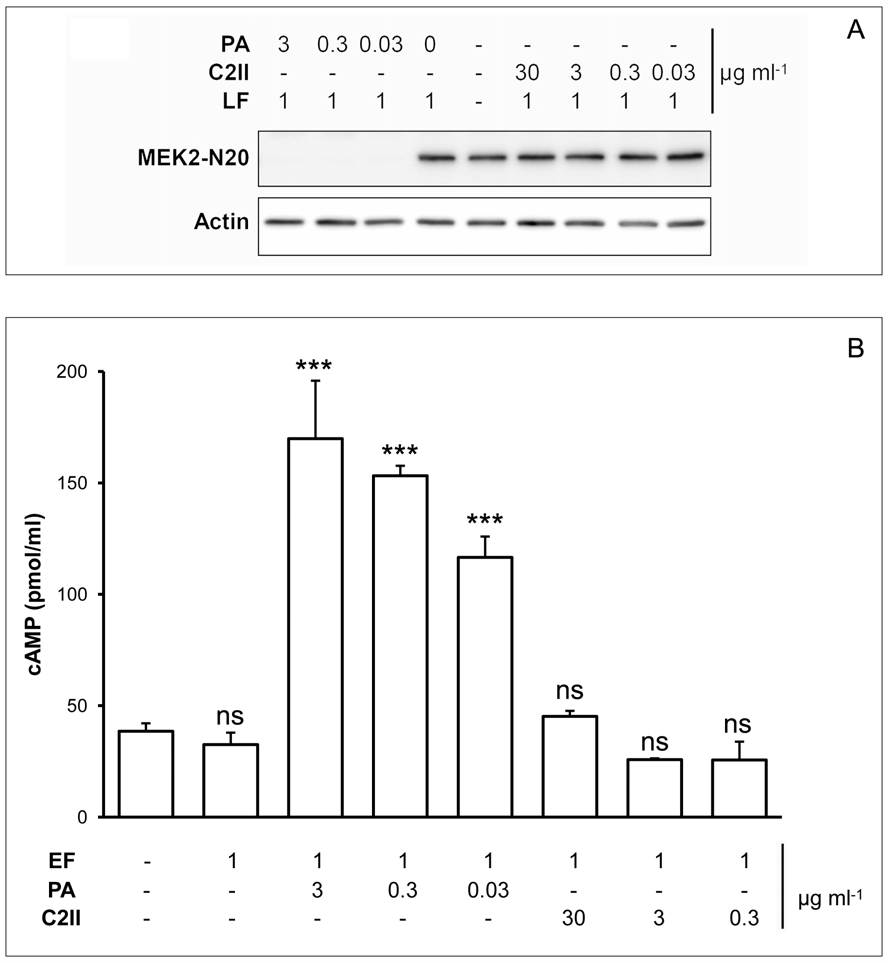
HUVECs intoxication by EF and LF using C2II in comparison to PA. All intoxication experiments were performed on HUVEC monolayers. Cells were treated overnight with either 1 µg/ml of LF or EF in the presence or absence of different amounts of PA or C2II, as indicated. **A**: Immunoblot anti-MEK2 showing the effect of MEK2 proteolysis by LF. 30 µg of total protein lysate were resolved on 12% SDS-PAGE. Anti-beta-actin immunoblot shows protein loading. MEK2 signals were normalized to actin. **B**: Graph shows measure of cyclic AMP (cAMP) cellular concentrations, expressed as pmol/ml. Mean values of two independent experiments ± SEM (ns: non significant and * p<0.05).

The enzymatic activity of the lethal factor (LF) of anthrax toxin can be measured by monitoring the cleavage of MAPKK, e.g. with MEK2 amino-terminal antibodies (anti-MEK2) [4]. HUVEC monolayers were intoxicated overnight with different combinations of PA-LF or C2II-LF and the activity of LF was analyzed on cell lysates by anti-MEK2 immunoblotting. Control experiments were performed in the absence of binding components. Whereas the wild-type lethal toxin (PA-LF) did not give any MEK2 signal after blotting, the combination of LF with different quantities of C2II revealed a defined signal of intact MEK2 (Figure 3A). Considering our findings that C2II mediated an efficient translocation of C2I into cells under these conditions (Figure 2A and 2B) we can present evidence that C2II has a dramatically lower capacity to promote translocation of LF into target cells.

Anthrax edema factor (EF) is known to increase cAMP in target cells when applied with its native binding partner PA_63_ [39]. We next tested whether C2II is able to promote translocation of EF by measure of the intracellular concentration of cAMP after overnight incubation of HUVEC monolayers. The combination of PA with EF led to a significant increase of intracellular cAMP level as a function of PA concentration. In contrast, the application of C2II-EF did not increase cAMP cellular levels significantly (Figure 3B). Even at very high concentration of C2II (30 µg/ml) the cellular cAMP level was approximately similar as the control and considerably below the effect of 0.03 µg/ml PA combined with 1 µg/ml EF. This means that translocation of EF by C2II is at least 1000-fold less efficient than PA when the same concentrations of PA and C2II were compared.

**Figure 4:**
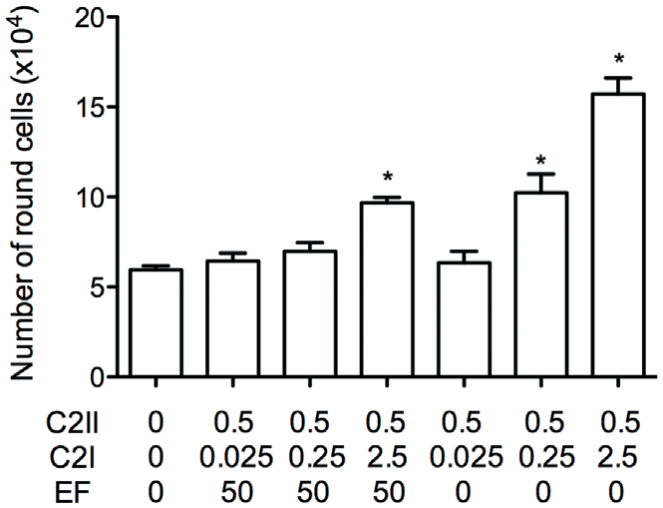
EF-mediated block of C2I-C2II intoxication *in vivo* (cell-based assay. EF-mediated inhibition of C2II-promoted C2I uptake into HUVEC cells. Cells were intoxicated with different concentrations of the binding component C2II and the effectors EF and C2I, as indicated, and the number of intoxicated cells was directly assessed by counting round cells. One representative experiment showing mean values of 5 independent counting for each condition.

We next verified that addition of EF was able to compete with C2I binding to C2II. The results are summarized in Figure 4. At a concentration of 50 µg/ml EF could significantly block C2II-mediated transport of C2I into HUVECs. These data further suggest that EF binds to the C2II channel *in vivo* (cell-based assay). Together, these findings show that C2II does not promote cell intoxication by EF and LF efficiently.

## Discussion

In previous studies we already demonstrated that the enzymatic components EF and LF of anthrax toxin bind to their B component protective antigen (PA_63_) and C2I of C2 toxin binds to its B component C2II *in vitro* [18], [20], [34]. PA shares significant sequence homology (35%) with C2II, indicating that the two proteins have similar modes of action. PA_63_ has been crystallized in its monomeric and heptameric prepore form [8] and a model of the C2II prepore structure has been constructed based on the corresponding assembly of the protective antigen prepore [21]. The similarity of both structures supports the view of a common mode of action, including the assumption that the enzymes bind in the vestibule of the channels of the corresponding binding component.

The results presented here suggest an interesting cross-over reactivity of anthrax and C2 toxins, despite a completely different primary and 3D-structure of the enzymatic compounds EF, LF and C2I [40], [41], [42]. The stability constants *K* for binding in the cross-over experiments *in vitro* were generally smaller than those for the native combinations, except the combination of C2II-EF. However, EF, LF and C2I show a high stability constant *K* for binding to PA_63_ and C2II heptamers in the cross-over experiments as the half saturation constants *K_s_* are between 2 and about 200 nM (see Table 1). These results refer to a common mechanisms and binding motifs within the enzymes' primary structures, in particular within the first three hundred amino acids of EF, LF and C2I. Truncated forms of EF and LF, called EF_N_ and LF_N_, bind with high affinity to the PA_63_ channels and support the transport of other polypeptides into target cells [26], [30], [43], [44], although the binding affinity of EF_N_ and LF_N_ for the PA_63_ channel is substantially reduced as compared to wild-type EF and LF [45]. Similarly, the N-terminal part of C2I is sufficient for transport of truncated forms of C2I and chimera proteins between the N-terminal end of C2I and other proteins into target cells [46], [47]. This means that the N-terminal ends of all enzymatic compounds interact with the PA_63_ and the C2II pores. Some of the amino acids responsible for these interactions are well known within the primary sequence of PA_63_ and its water-soluble prepore, e.g. amino acids E398, D425 and F427 (also known as the Φ-clamp) [22], [48], [49], but relatively unknown for C2II, although there exist some indications that the corresponding amino acids E399, D426 and F428 may play a similar role for C2I binding [50]. However, further amino acids responsible for this interaction still need to be identified.

The amino acids responsible for binding within the N-terminal end of the enzymatic components are relatively unknown, although there is clear evidence that positively charged amino acids are involved as they form salt bridges between the enzymatic components and the channels. The positively charged N-termini of the enzymes is presumably decisive as quaternary ammonium ions and 4-aminoquinolones show a blockage of PA_63_ and C2II channels in lipid bilayer experiments [34], [36], [51], [52]. The selectivity of the two channels for cations, which is at least partially due to the charged amino acids in the β-barrel, may also play a significant role. Both channels are known to prefer cations over anions in zero-current membrane-potentials, the *P*_cation_ over *P*_anion_, as described by the Goldman-Hodgkin-Katz equation [53], are 20 for PA_63_ channels and 10 for C2II channels, respectively [54], [55]. Therefore cations have a strong effect on the single channel conductance as compared to anions [54], [55]. It may be possible that the differently charged channel interiors of PA and C2II have a decisive influence on binding and transport of the enzymatic components (see below). Altogether there exist strong indications that binding to the different channels follows different mechanisms.

Another conceivable possibility is that the structure of the channel itself is important for translocation. The extended channel-forming β-sheets of the PA_63_ monomers contain three glutamic acids and three aspartic acids (E302, E308, E343 and D276, D315, D335), so the extended β-barrel could contain up to 48 negatively charged groups, which probably cannot be counterbalanced by the at least partially positively charged histidines H304 and H310 [56], [57], [58]. However, the C2II channel contains 7 glutamic acids (E307) and 14 histidines (H296 and H332), indicating that it has a much smaller overall charge [31]. The interaction of the charged groups of the channel interior and the bound enzymatic components could be different for channels leading to divergent uptake efficiency. Considering the fact that the charges in the vestibule domain are quite balanced in both PA_63_ and C2II, i.e. both have 14 acidic amino acids facing the interior of the vestibule domain; the effect of the charges in the water filled β-barrels should be striking. As mentioned beforehand, the C2II channel is missing most of them.

The most interesting result of this study was that the combination of PA with C2I showed HUVEC toxicity. This appeared specific of PA considering the rather poor capacity of C2I to bind and to trigger cell intoxication by LF. This clearly reveals that PA has the remarkable ability to bind and to translocate an enzymatic component of another AB_7/8_ type toxin into cells. The level of cell intoxication with C2I via PA, however, was at least 50-fold less efficient than with the wild-type combination C2II-C2I. We can only speculate about the reasons of this higher flexibility of PA as compared to C2II. One possibility is that a different driving force is required to translocate EF and LF through the C2II channel because EF and LF are released at the state of the late endosome [9], whereas C2I leaves the early endosome following acidification and, in addition, depends on the help of the cytosolic chaperon Hsp90 [16], [59]. A similar requirement is not known for the translocation of LF, EF or LF's N-terminal domain (LF_N_) through the PA_63_ channel, where a pH-gradient across the membrane creates a sufficient driving force for translocation of the proteins [24]. With the evidence presented here, i.e. that some components of the highly specialized binary toxins can be interchanged without loss of toxicity, further work with mutated binding components, enzymatic moieties and chimeras seems to be necessary to understand the different translocation capacities of PA_63_ and C2II channels.
